# Characterization of Ultrasound Energy Diffusion Due to Small-Size Damage on an Aluminum Plate Using Piezoceramic Transducers

**DOI:** 10.3390/s17122796

**Published:** 2017-12-04

**Authors:** Guangtao Lu, Qian Feng, Yourong Li, Hao Wang, Gangbing Song

**Affiliations:** 1 Key Laboratory for Metallurgical Equipment and Control of Ministry of Education, Wuhan University of Science and Technology, Wuhan 430081, China; luguangtao@wust.edu.cn; 2 Key Laboratory of Earthquake Geodesy, Institute of Seismology, China Earthquake Administration, Wuhan 430071, China; fengqian@eqhb.gov.cn (Q.F.); wanghao@eqhb.gov.cn (H.W.); 3 Hubei Key Laboratory of Mechanical Transmission and Manufacturing Engineering, Wuhan University of Science and Technology, Wuhan 430081, China; liyourong@wust.edu.cn; 4 Smart Materials and Structures Laboratory, Department of Mechanical Engineering, University of Houston, Houston, TX 77204, USA

**Keywords:** piezoceramic transducers, ultrasound energy diffusion, small size damage, Lamb waves, damage detection, structural health monitoring

## Abstract

During the propagation of ultrasonic waves in structures, there is usually energy loss due to ultrasound energy diffusion and dissipation. The aim of this research is to characterize the ultrasound energy diffusion that occurs due to small-size damage on an aluminum plate using piezoceramic transducers, for the future purpose of developing a damage detection algorithm. The ultrasonic energy diffusion coefficient is related to the damage distributed in the medium. Meanwhile, the ultrasonic energy dissipation coefficient is related to the inhomogeneity of the medium. Both are usually employed to describe the characteristics of ultrasound energy diffusion. The existence of multimodes of Lamb waves in metallic plate structures results in the asynchronous energy transport of different modes. The mode of Lamb waves has a great influence on ultrasound energy diffusion as a result, and thus has to be chosen appropriately. In order to study the characteristics of ultrasound energy diffusion in metallic plate structures, an experimental setup of an aluminum plate with a through-hole, whose diameter varies from 0.6 mm to 1.2 mm, is used as the test specimen with the help of piezoceramic transducers. The experimental results of two categories of damages at different locations reveal that the existence of damage changes the energy transport between the actuator and the sensor. Also, when there is only one dominate mode of Lamb wave excited in the structure, the ultrasound energy diffusion coefficient decreases approximately linearly with the diameter of the simulated damage. Meanwhile, the ultrasonic energy dissipation coefficient increases approximately linearly with the diameter of the simulated damage. However, when two or more modes of Lamb waves are excited, due to the existence of different group velocities between the different modes, the energy transport of the different modes is asynchronous, and the ultrasonic energy diffusion is not strictly linear with the size of the damage. Therefore, it is recommended that only one dominant mode of Lamb wave should be excited during the characterization process, in order to ensure that the linear relationship between the damage size and the characteristic parameters is maintained. In addition, the findings from this paper demonstrate the potential of developing future damage detection algorithms using the linear relationships between damage size and the ultrasound energy diffusion coefficient or ultrasonic energy dissipation coefficient when a single dominant mode is excited.

## 1. Introduction

Structural health monitoring (SHM) has been receiving an increasing amount of attention in recent years [1,2,3,4,5,6,7]. For damage detection in an SHM system, a PZT (lead zirconate titanate) transducer is usually utilized as an actuator to generate ultrasonic waves along a structure, and other PZT transducers are used as sensors to detect the changes in both environmental and operational conditions [8,9,10,11,12] and structural damages [13,14,15,16,17,18]. The damage-induced changes in properties such as electromechanical impedance [19,20,21,22,23,24,25,26,27,28], ultrasonic energy [29,30,31,32,33,34], nonlinear characteristics of Lamb waves [35,36,37,38], and other ultrasonic parameters [39,40], are further employed to estimate the health state of the structure [41,42,43,44,45,46] or locate the damage [47,48,49,50,51]. However, previous studies of damage detection mainly focus on how to make use of these structural changes to estimate or locate the damage, but do not consider the ultrasonic changes which are brought about by structural damage.

In fact, the interaction between structural damage and ultrasonic changes can be explained by ultrasound energy diffusion [52,53,54]. Studies of ultrasound energy diffusion indicate that the change of ultrasound energy is associated with energy losses due to energy diffusion and energy dissipation. They also indicate that the ultrasound energy diffusion is mainly related to the structures, including damages distributed in the medium, while ultrasonic energy dissipation is mainly caused by energy absorption due to the inhomogeneity of the medium [52,53,54,55,56,57]. Weaver [52,55] theoretically studied the characteristics of ultrasound energy diffusion in an aluminum foam and polycrystals, and established a mathematical model to reveal the relationship between the ultrasonic diffusion energy and microstructure. Weaver’s study was validated by experimental investigation and numerical simulation, which were carried out by Anugonda et al. [53] and Schubert and Koehler [54], respectively. In recent years, applications of ultrasound diffusion to detect damage detection in concrete structures were also developed [58,59,60,61,62].

However, the aforementioned studies of ultrasound energy diffusion mainly involve concrete structures; little attention is paid to ultrasound energy diffusion in metallic structures, especially in thin plate-like structures. Since the scattering characteristics of the waves in concrete structures are much stronger than those in metallic structures, the effect of ultrasound energy diffusion will be much weaker in a metallic structure. Moreover, due to the existence of dispersion and multimodes of Lamb waves [63,64,65,66,67], different modes of Lamb waves propagate in the structure with different group velocities, and the energy of different modes is transported asynchronously. This makes the characteristics of ultrasound energy diffusion in thin plate-like structures more complicated. Therefore, the characteristics of ultrasound energy diffusion in metallic plate structures need further investigation.

In this paper, the characteristics of ultrasound energy diffusion in an aluminum plate due to small damages are studied with the help of piezoceramic transducers. The existence of multimodes of Lamb waves in metallic plate structures results in the asynchronous energy transport of different modes. A mode of Lamb waves has a great influence on the ultrasound energy diffusion, and thus needs to be chosen appropriately. In this research, both single dominant modal excitation and multimodal excitation are conducted. With the excitation of only one dominate mode of Lamb wave in the plate structure, the ultrasound energy diffusion coefficient decreases approximately linearly with the diameter of the damage, while the ultrasonic energy dissipation coefficient increases approximately linearly with the diameter of the simulated damage. However, when two or more modes of Lamb waves are excited, due to the existence of different group velocities between different modes, the energy transport of different modes is asynchronous, and the ultrasonic energy diffusion is not strictly linear with the size of the damage. Therefore, it is recommended that only one dominant mode of Lamb wave should be excited during the characterization process in order to ensure the linear relationship between the damage size and the characteristic parameters. We hope that the findings in this study can offer guidelines for the future development of damage detection algorithms based on ultrasonic energy diffusion.

The rest of the paper is organized as follows. In Section 2, the fundamentals of ultrasound energy diffusion are presented, and the process of computing the ultrasound energy diffusion is developed. In Section 3, the influence of the dispersive characteristics of the Lamb wave on the ultrasound energy diffusion is analyzed. In Section 4, an experimental setup of an aluminum plate with a through-hole, whose diameter is varied from 0.6 mm to 1.2 mm, is designed to characterize the ultrasound energy diffusion. The experimental results are analyzed and discussed in Section 5. At last, Section 6 concludes the paper and suggests areas for future work.

## 2. Fundamentals of Ultrasound Energy Diffusion

Based on the theory of ultrasound energy diffusion, the spectral energy density describes the energy diffusion of an ultrasonic wave, and the spatiotemporal evolution of spectral energy density $E(\vec{r},t)$ can be given by the ultrasound energy diffusion equation [52,68,69],

(1) $$\frac{\partial E(\vec{r},t)}{\partial t}+D\nabla^{2}E(\vec{r},t)-\sigma E(\vec{r},t)=E_{0}\delta (\vec{r}-{\vec{r}}_{0})\delta (t-t_{0})$$

where *D* is the frequency-dependent ultrasound energy diffusion coefficient, which is related to the distribution of damage in the medium; *σ* is the frequency-dependent ultrasonic energy dissipation coefficient, which is related to the inhomogeneity of the medium; *E*_0_ is the initial energy, which is stored at point ${\vec{r}}_{0}$ at time *t*_0_; and *δ* is the Dirac delta function. It should be emphasized that the ultrasound energy diffusion coefficient *D* is influenced by the microstructure, and the ultrasonic dissipation energy coefficient σ is mainly influenced by the material of the structure. Therefore, the ultrasound energy diffusion coefficient *D* and ultrasonic energy dissipation *σ* can be employed to identify the small-size damage in a structure.

The series solution to Equation (1) for a three-dimensional structure is given by Equation (2) [57,58,59],

(2) $$\begin{array}{cc} E(x,y,z,t) & =E_{0}e^{-\sigma t}\{1+g(x,x_{0},a)g(y,y_{0},b)g(z,z_{0},c)\\ & +\left[g(x,x_{0},a)+g(y,y_{0},b)+g(z,z_{0},c)\right]\\ & +g(x,x_{0},a)g(y,y_{0},b)\\ & +g(x,x_{0},a)g(z,z_{0},c)\\ & +g(y,y_{0},b)g(z,z_{0},c)\}E_{0}e^{-\sigma t} \end{array}$$

where $g(X,X_{0},A)=\sum_{m=1}^{\infty }2\cos(m\pi \frac{X_{0}}{A})\cos(m\pi \frac{X}{A})e^{-Dt{\left(\frac{m\pi }{A}\right)}^{2}}$, *a*, *b*, and *c* are dimensions of the structure, (*x*_0_, *y*_0_, *z*_0_) indicates the location of ultrasonic actuator, and (*x*, *y*, *z*) indicates the location of sensor where the energy is measured.

Furthermore, for an infinite one-dimensional structure, the solution to Equation (1) is expressed as [53],

(3) $$E(x,t)=E_{0}\frac{1}{2\sqrt{\pi Dt}}e^{-\frac{x^{2}}{4Dt}}e^{-\sigma t}$$

where *E*_0_ is the initial energy stored at the excitation point.

Equations (2) and (3) indicate that the ultrasound energy density is related to the locations of the energy input and the energy receiver, as well as the structural dimension. These relationships are distinct from those for the ultrasound energy diffusion coefficient and the ultrasound energy dissipation coefficient, which are only related to the medium. If the distance between the actuator and the sensor changes, or the dimension of the structure changes, the ultrasound energy density will also change. However, the ultrasound energy diffusion coefficient and ultrasound energy dissipation coefficient would not be influenced.

The logarithmic form of Equation (3) is

(4) $$\ln E(x,t)=C_{0}-\frac{x^{2}}{4Dt}-\sigma t-0.5\ln(Dt)$$

where *C*_0_ is a constant that is related to the initial energy.

For a given structure, the constant *C*_0_, the ultrasound energy diffusion coefficient *D* and the ultrasonic energy dissipation *σ* in Equation (4) can be determined by curve fitting using the experimental data, and the detailed procedure is listed below:

(1) divide the time-domain signal into short segments by a time window of a length of *Δt* with a specific window overlap ratio *γ*,
(2) compute the spectral power of each segment by discrete-time Fourier transform (DTFT),
(3) compute the spectral energy density by summing up the power spectrum of each segment in a specific bandwidth *Δf* centered at frequency *f_c_*, and
(4) determine the ultrasound energy diffusion coefficient D and ultrasonic energy dissipation σ by curve fitting.

## 3. Dispersive Characteristics of Lamb Waves

Based on the theory of ultrasound energy diffusion, when the wavelength is on the order of the size of the damages, the ultrasonic waves will be greatly influenced by the multiple scattering effects due to the damages, and the ultrasonic signals can be separated into diffusive and dissipative components. The dispersive characteristics of the Lamb wave will affect the ultrasound energy diffusion.

According to the principles of Lamb waves, Lamb waves propagate in thin plate-like structures with two parallel free boundaries, and the motion of Lamb waves is the resultant motion of antisymmetric motion A*_i_* and symmetric motion S*_i_* (*i* = 0, 1, 2 …), which are expressed by Rayleigh-Lamb equations [70]

(5) $$\{\begin{array}{c} \mathrm{Symmetric}\;\mathrm{motion}:\;\frac{\tan(qh)}{\tan(ph)}=-\frac{4k^{2}qp}{{\left(k^{2}-q^{2}\right)}^{2}}\\ \mathrm{Antisymmetric}\;\mathrm{motion}:\;\frac{\tan(qh)}{\tan(ph)}=-\frac{{\left(k^{2}-q^{2}\right)}^{2}}{4k^{2}qp} \end{array}$$

where $p^{2}=\omega^{2}/c_{L}^{2}-k^{2}$, $q^{2}=\omega^{2}/c_{S}^{2}-k^{2}$, λ=2π/k, ω=2πf, $c_{L}^{2}=\frac{2\mu (1-\nu )}{\rho (1-2\nu )}$, $c_{S}^{2}=\mu /\rho$. In addition, h is the half-thickness of the plate; *μ*, *ν,* and *ρ* are the shear modulus, Poisson’s ratio, and mass density of the medium, respectively; *λ*, *k*, and f are the wavelength, wavenumber, and frequency of the waves, respectively; and *c_S_* and *c_L_* are the transverse and longitudinal velocities of the waves, respectively. Equation (4) indicates that, for a given structure, both the group velocity $c_{i}=\omega /k_{i}$ and wavelength *λ* change with the frequency *f*, and this change of group velocity with frequency causes the Lamb waves to be highly dispersive.

**Figure 1:**
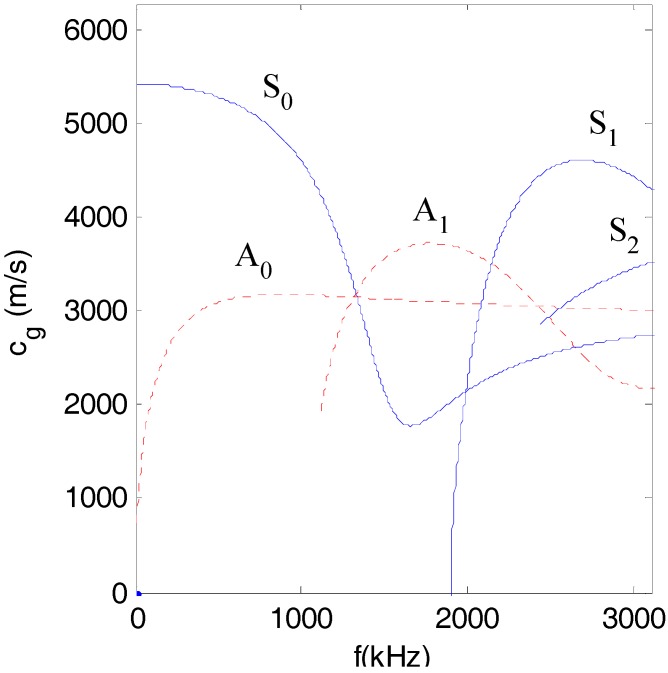
Dispersive curve of group velocity (Alloy 5052, half-depth *h* = 0.75 mm).

Figure 1 plots the dispersive curves of the group velocity of Lamb waves for a given structure (half-depth *h* = 0.75 mm). It can be seen from Figure 1 that for a given structure, the group velocities of different modes at a certain frequency are different, which means that the ultrasonic energy that is transported by different modes of Lamb waves is asynchronous. Therefore, in order to obtain a strong effect of ultrasound energy diffusion, the modes of Lamb waves should be chosen carefully.

**Figure 2:**
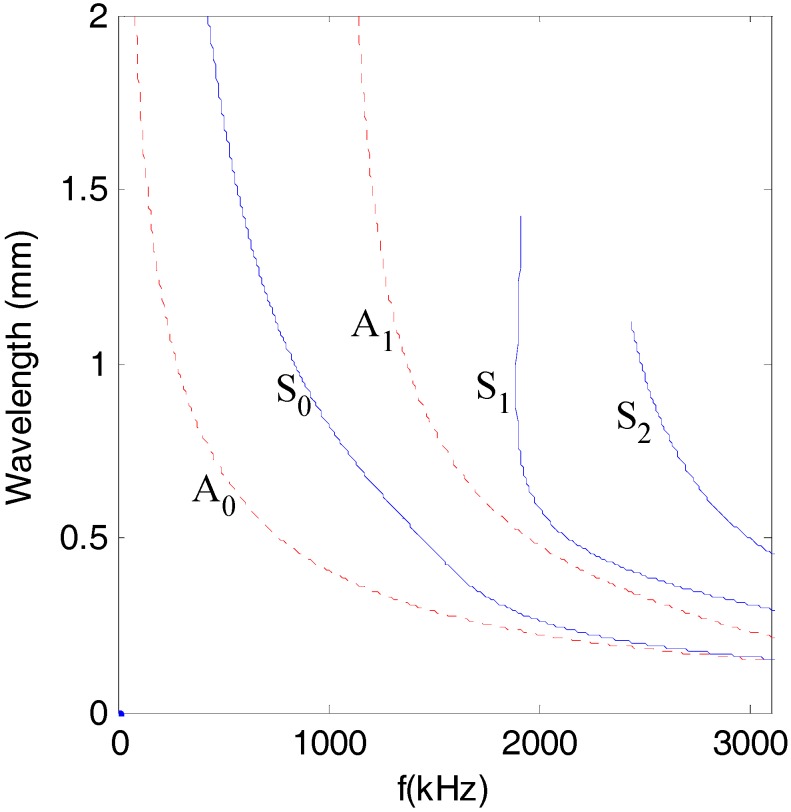
Dispersive curve of wavelength (Alloy 5052, half-depth *h* = 0.75 mm).

In addition, for the purpose of easily separating the diffusive and dissipative components of the ultrasonic signals, the wavelengths of all of the modes of Lamb waves should be less than the maximum size of all the damages. Figure 2 plots the wavelength of Lamb waves for a given structure (half-depth *h* = 0.75 mm).

However, based on the tuned Lamb wave theory [71], when a PZT is attached to a given structure to excite Lamb waves, there are many tuned frequencies at which the displacement of one certain mode of Lamb wave is zero. Among these tuned frequencies, such frequencies where there is only one dominant mode of Lamb wave can be obtained by a numerical method.

## 4. Experimental Setup

**Figure 3:**
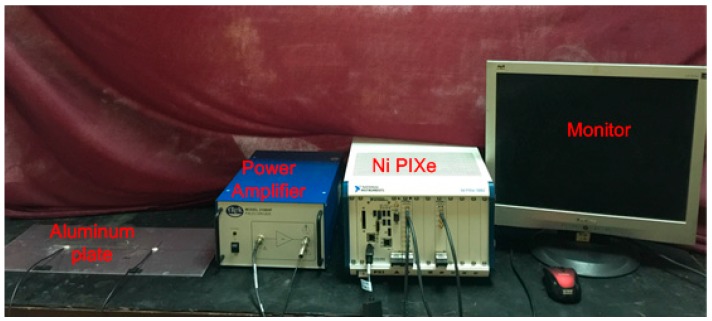
The experimental setup with the data acquisition system.

**Figure 4:**
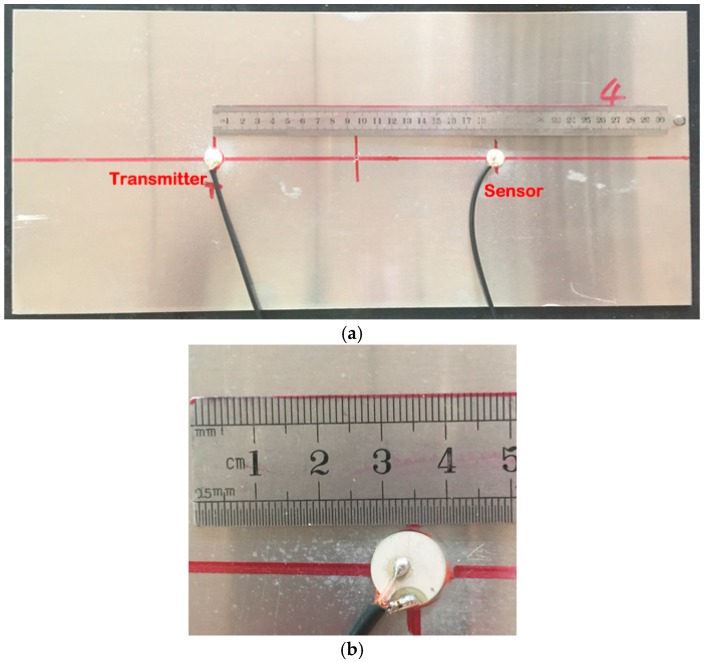
An aluminum plate with PZT (lead zirconate titanate) disks: (**a**) an aluminum plate (Alloy 5052) with a PZT transmitter and a PZT sensor; (**b**) a PZT on the aluminum plate.

To investigate the characteristics of the ultrasound energy diffusion of Lamb waves, an experimental setup is designed and fabricated, as shown in Figure 3. The experimental setup mainly consists of an aluminum plate (Alloy 5052) bonded with two PZT disks (shown in Figure 4), an amplifier with a bandwidth of 0–3.0 MHz and a fixed gain of 50 (Trek Model 2100H) for a piezoceramic load, a signal generation, a data acquisition system (a Ni PXIe-1082 chassis with a Ni PXI-5412 arbitrary waveform generator and a Ni PXIe-5105 digitizer), and a monitor.

**Figure 5:**
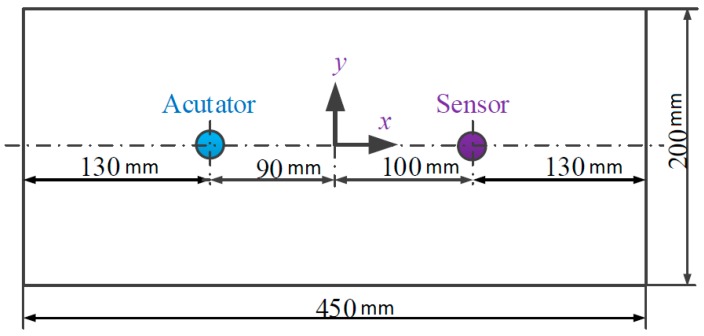
Structural parameters of the aluminum plate.

**Figure 6:**
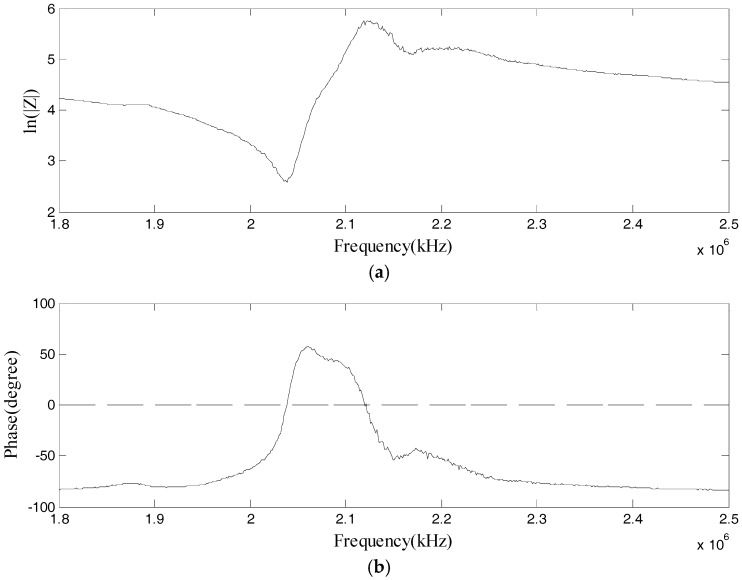
Impedance of the bonded PZT disk: (**a**) magnitude of the impedance; (**b**) phase angle of the impedance.

Since the ultrasound energy diffusion coefficient and the ultrasound energy dissipation coefficient are not influenced by the locations of the actuator and the sensor nor the dimension of the plate, an aluminum plate with a dimension of 450 mm × 200 mm × 1.5 mm is selected, and as shown in Figure 5, two PZT disks are bonded on the plate by epoxy at two fixed locations. The size of the PZT disk is Φ12 mm × 1 mm, and the locations of these two PZTs are also shown in Figure 6. During the experiments, one of the two PZTs is chosen as an actuator, and the other is used as a sensor.

To investigate the relationship between the size of the damage and the ultrasound energy diffusion coefficients, two different damage categories are tested, and for each category, the simulated damage is a through-hole with different center locations. For each category, three specimens are tested. The locations of the simulated damages are listed in Table 1. For each damage category, the diameter of the hole is increased from 0.6 mm to 1.2 mm with an increment of 0.2 mm. Therefore, there are 10 different damages in total, and three different excitation pulses are employed to excite Lamb waves for each damage.

**Figure 7:**
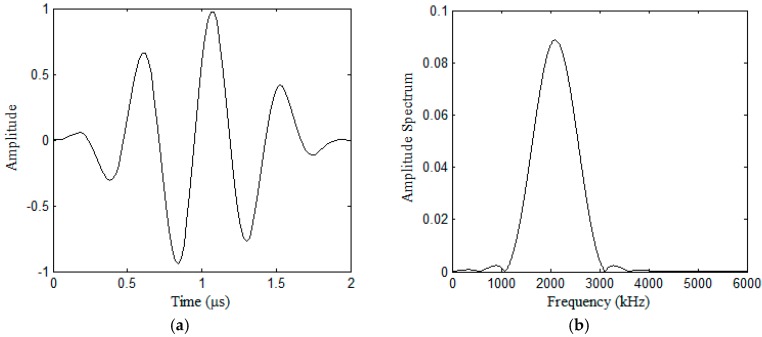
Excitation pulse in the time and frequency domains: (**a**) time domain; (**b**) frequency domain.

To obtain the maximum output from the actuator, the excitation frequency of the pulse should be near the resonant frequency of the bonded PZT disk. The electrical impedance of the PZTs is measured by using an impedance analyzer (Wayne Kerr 6500B), and the impedance curve is shown in Figure 6. In addition, considering that the wavelengths of all of the modes of Lamb waves should be less than the maximum size of all of the damages, in order to study the influence of the dispersive characteristics of Lamb waves on the ultrasound energy diffusion, the center frequency of the excitation pulse is selected as 1.8~2.4 MHz, and the pulse is a Hanning-windowed tone burst with a peak amplitude of 2.8 volts. During the experiments, the Ni PXI-5412 arbitrary waveform generator is used to generate the pulse at a sampling frequency of 50 MHz. The pulse is then sent to the actuator after it is amplified, and the signal is recorded by the Ni PXIe-5105 digitizer at a sampling frequency of 60 MHz. The total sampling time is 1000 µs. Figure 7 shows the excitation pulse in the time and frequency domains. All of the experiments were conducted in the same room with an environmental temperature of 30 °C, and all of the data was measured with environmental noise.

## 5. Experimental Results

After the temporal signals were obtained, they were further processed to obtain the ultrasonic energy density, the ultrasound energy diffusion coefficient, and the ultrasonic energy dissipation coefficient.

To decrease the adverse effect of the epoxy between the aluminum and the PZT on the location error of the PZTs, the ultrasound energy diffusion coefficient and the ultrasonic energy dissipation coefficient are normalized by the value when there is no damage in the aluminum plate.

**Figure 8:**
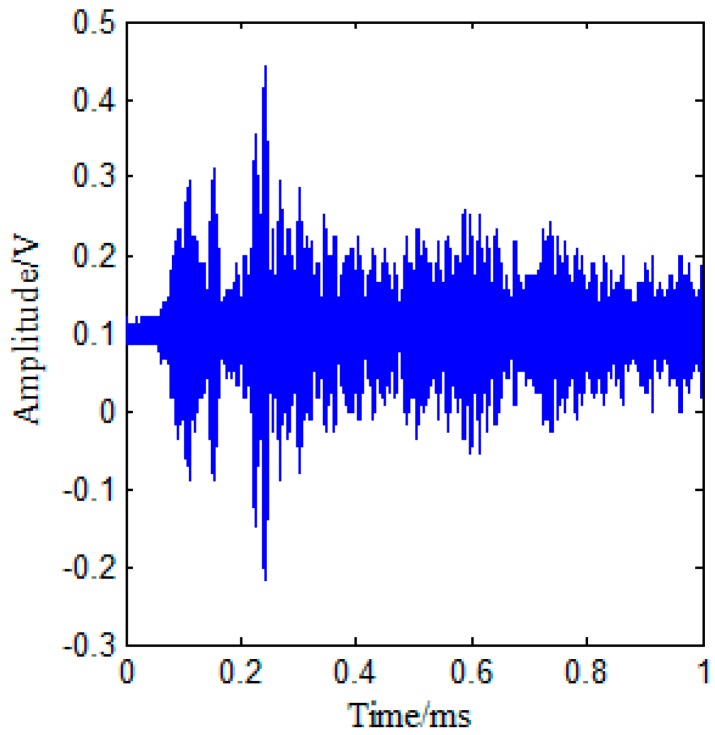
Recorded signal with environmental noise.

**Figure 9:**
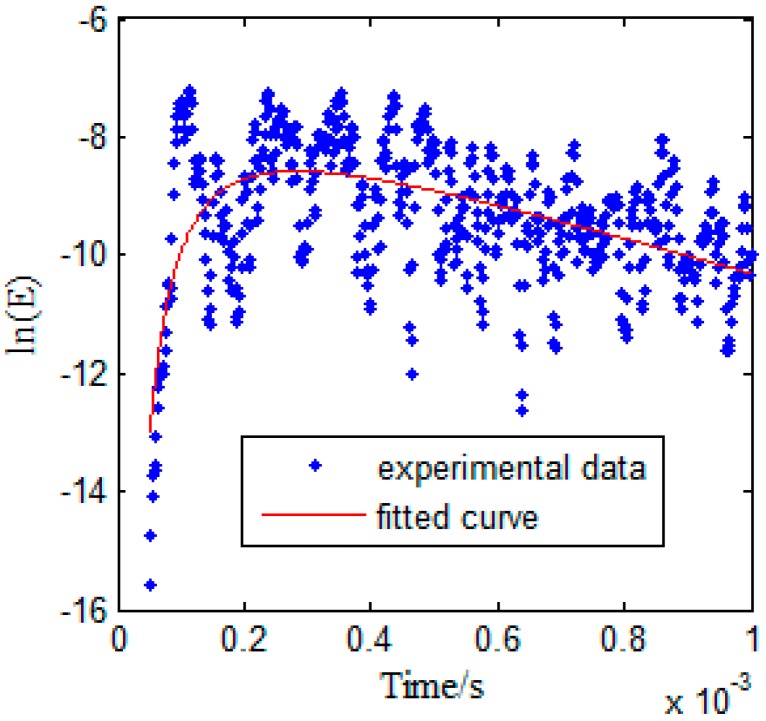
Ultrasonic energy density curves of Lamb waves with different damages under an excitation pulse with a center frequency of *f* = 2.1 MHz, when there is no damage.

Figure 8 shows the measured temporal signal with environmental noise. The parameters for signal processing described in Section 2 are selected as Δ*t* = 16.67 μs, *γ* = 0.9, Δ*f* = 0.15 MHz, *f_c_* = 2.1 MHz, and the total recorded time *t* = 1 ms. The ultrasonic energy density is then obtained by the steps listed in Section 2. The ultrasound energy density curve under an excitation pulse with a center frequency of *f* = 2.1 MHz (when there is no damage) is plotted in Figure 9, and the red curve in Figure 9 is obtained by curve fitting, based on Equation (4).

### 5.1. Influence of Damage Size on Ultrasound Energy Diffusion

According to Figure 6, one resonant frequency of the PZT patch is about 2.1 MHz, which was therefore selected as the central frequency for the excitation pulse. With this excitation, the responses of the plate with damage categories D1 and D2 were recorded. Table 2, Table 3, Table 4 and Table 5 respectively list the normalized ultrasound energy diffusion coefficients of damage categories D1 and D2. Table 2 and Table 3 reveal that the normalized ultrasound energy diffusion coefficient monotonously decreases from 94.71% to 91.46% when the diameter of damage D1 increases from 0.6 mm to 1.2 mm, while the normalized ultrasonic energy dissipation coefficient monotonously increases from 103.92% to 114.79%. Table 4 and Table 5 show that the normalized ultrasound energy diffusion coefficient monotonously decreases from 96.46% to 94.24% when the diameter of damage D2 increases from 0.6mm to 1.2 mm, while the normalized ultrasonic energy dissipation coefficient monotonously increases from 103.84% to 107.02%.

However, compared with the dimensions of the aluminum plate, the volume proportion of the through-hole is still very small, even though the diameter of the through-hole increases from 0.6 mm to 1.2 mm, and therefore the relative change of ultrasound energy density is small compared with the relative change of the diameter of the simulated hole.

**Figure 10:**
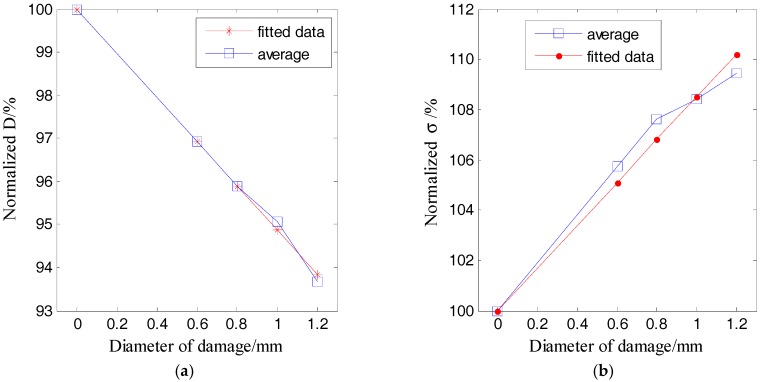
Normalized ultrasonic energy coefficients of damage D1 versus the damage size: (**a**) ultrasound energy diffusion coefficient; (**b**) ultrasonic energy dissipation coefficient.

**Figure 11:**
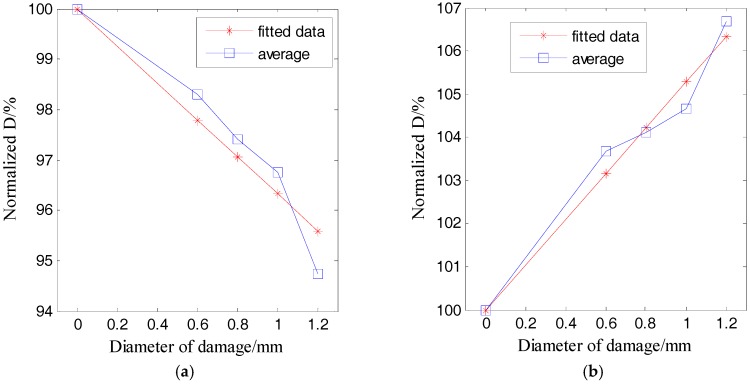
Normalized ultrasonic energy coefficients of damage D1 versus the damage size: (**a**) ultrasound energy diffusion coefficient; (**b**) ultrasonic energy dissipation coefficient.

Figure 10 and Figure 11 respectively plot the ultrasound energy coefficients versus the damage size for the damage cases of D1 and D2.

Both Figure 10 and Figure 11 demonstrate that the average of the ultrasound energy diffusion coefficient decreases approximately linearly with the diameter of the through-hole damage, while the ultrasonic energy dissipation coefficient increases approximately linearly with the diameter of the same damage.

### 5.2. Influence of Dispersion of Lamb Waves on Ultrasound Energy Diffusion

As discussed in Section 3, due to the existence of the dispersions of Lamb waves, the propagation characteristics of Lamb waves are complicated. Therefore, the influence of the dispersion of Lamb waves on the ultrasound energy diffusion is also investigated.

**Figure 12:**
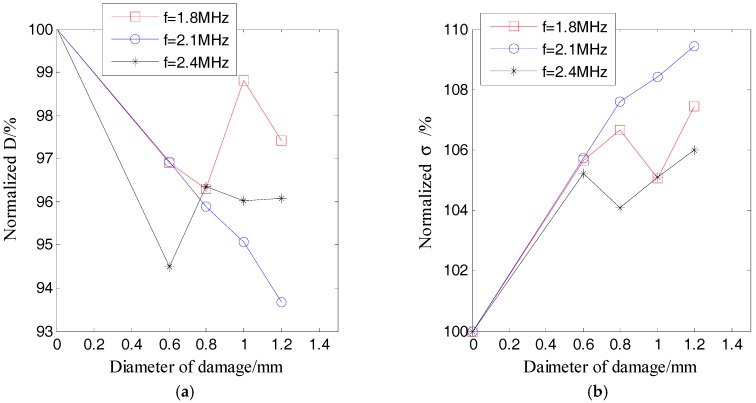
Normalized ultrasonic energy coefficients of damage D1 versus the damage size under different excitation pulses: (**a**) ultrasound energy diffusion coefficient; (**b**) ultrasonic energy dissipation coefficient.

**Figure 13:**
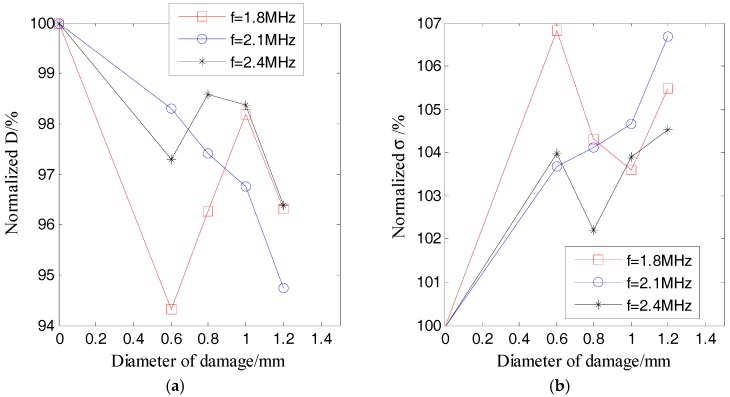
Normalized ultrasonic energy coefficients of damage D2 versus the damage size under different excitation pulses: (**a**) ultrasound energy diffusion coefficient; (**b**) ultrasonic energy dissipation coefficient.

**Figure 14:**
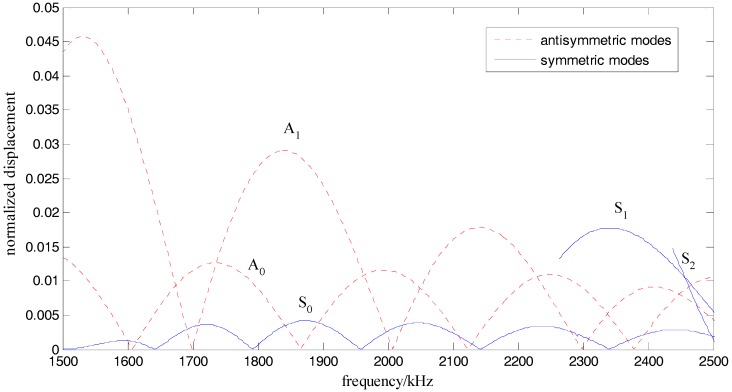
Normalized displacement of different modes of Lamb waves.

Figure 12 and Figure 13 respectively show the ultrasound energy coefficients of damage category D1 under excitation pulses with different center frequencies. Figure 14 plots the normalized displacements of different modes of Lamb waves, which are obtained based on the tuned Lamb wave theory [71]. Table 6 and Table 7 respectively list the normalized displacements and group velocities of different modes of Lamb waves at different frequencies.

Figure 12a and Figure 13a indicate that all of the normalized ultrasound energy diffusion coefficients in the presence of damage were less than those without damage. However, the changes of the normalized ultrasound energy diffusion coefficients under excitation pulses with center frequencies of *f* = 1.8 MHz and *f* = 2.4 MHz were no longer linear to the damage size.

Figure 12b and Figure 13b show that, in the presence of damage, all of the normalized ultrasonic energy dissipation coefficients were larger than those without damage. However, the changes of the normalized ultrasonic energy dissipation coefficients under excitation pulses with center frequencies of *f* = 1.8 MHz and *f* = 2.4 MHz were also no longer linear to the damage size.

As shown in Figure 14 and listed in Table 6, when the center frequency was 1.8 MHz, the normalized displacement of the A_0_ mode was about 1/3 of that of the A_1_ mode. When the center frequency was 2.1 MHz, the normalized displacement of the S_0_ mode and the A_0_ mode were about 1/7 and 1/5 of that of the A_1_ mode, respectively. When the center frequency was 2.4 MHz, the normalized displacement of the A_0_ mode was about 9/16 of that of the S_1_ mode. Therefore, when the excitation frequencies were 1.8 MHz and 2.4 MHz, which are both far from the tuned frequency, there were mainly two modes (A_0_ and A_1_ modes for 1.8 MHz, S_1_ and A_0_ modes for 2.4 MHz), which transported most of the energy (the energy was proportional to the power of the displacement) of the excitation pulse. On the contrary, when the frequency was 2.1 MHz, which is near to the tuned frequency, there was mainly only one mode (A_1_ mode) in the plate. In addition, after considering the group velocities listed in Table 7, when the frequencies were 1.8 MHz and 2.4 MHz, respectively, the differences of the group velocities of the two dominant modes were 625 m/s and 1339 m/s, respectively. These results indicated that the energy transport of these two dominant modes was asynchronous, and therefore, the ultrasound energy diffusion is not strictly linear to the size of the damage when the center frequency of the excitation is 1.8 MHz and 2.4 MHz.

Please note that a change in the ultrasound energy diffusion can still be used to detect the existence of damage, since there is a distinct change in the ultrasound energy diffusion when there is damage.

### 5.3. Discussion

As illustrated in the above sections, the ultrasonic energy diffusion changes if there is damage to the structure, and the dispersion and multimode characteristics of Lamb waves have a large influence on the diffusion of the ultrasound energy. When there is only one dominant mode of Lamb wave in the structure, the ultrasound energy diffusion coefficients have a linear relationship with the diameter of the through-hole damage. However, when there are two or more modes of Lamb waves, due to the existence of different group velocities between the different modes, the energy transport of the different modes is asynchronous, and the ultrasonic energy diffusion is not strictly linear with the size of the damage.

Therefore, it is recommended that only one dominant mode of Lamb wave should be excited during the characterization process in order to ensure that the linear relationship between the damage size, the characteristic parameters, and the optimal frequency can be computed in advance of the damage through a numerical method based on the tuned Lamb wave theory. In addition, the findings from this paper demonstrate the potential for future damage detection algorithms to be developed using the linear relationships between damage size and the ultrasound energy diffusion coefficient or ultrasonic energy dissipation coefficient. When a single dominant mode is excited, structural damage detection can be carried out by two main steps: (1) obtaining the location of the damage through developed approaches, such as the delay-and-sum imaging algorithm, and (2) detecting the size of the damage by using the linear relationship between the damage size, the ultrasound energy diffusion coefficient, and the ultrasound energy dissipation coefficient.

## 6. Conclusions

The characteristics of ultrasound energy diffusion in a metallic plate structure in the presence of a small through-hole are studied in this paper with the help of piezoceramic transducers. According to the theory of ultrasound energy diffusion, the propagation of ultrasonic waves in structures is usually associated with energy losses due to energy diffusion and energy dissipation. The ultrasound energy diffusion coefficient is related to the microstructure of the medium, the ultrasonic energy dissipation coefficient is related to the inhomogeneity of the medium, and both coefficients are obtained by data fitting. To study the characteristics of ultrasound energy diffusion, an experimental setup with an aluminum plate with a through-hole was designed and fabricated. The experimental results of two categories of damages revealed that the presence of damage changed the energy transport between the actuator and the sensor, as well as both the ultrasonic energy diffusion coefficient and the ultrasonic energy dissipation coefficient. In addition, when there was only one dominant mode of Lamb wave excited in the structure, the average of the ultrasound energy diffusion coefficient decreased approximately linearly with the diameter of the through-hole damage, while the ultrasonic energy dissipation coefficient increased approximately linearly with the diameter of the through-hole damage. However, when two or more modes of Lamb waves were excited, due to the existence of difference of group velocities between the different modes, the energy transport of different modes was asynchronous, and the ultrasonic energy diffusion was not strictly linear with the size of the damage. Therefore, it is recommended that only one dominant mode of Lamb wave should be excited during the characterization process in order to ensure a linear relationship between damage size and the characteristic parameters.

Future work will involve the in-depth experimental study of the characteristics of ultrasound energy diffusion in a plate with complex structures and different materials, and damage detection based on ultrasound energy diffusion and its potential applications in structural health monitoring. For ultrasound energy diffusion-based damage size detection in a thin-plate-like structure, the center frequency of excitation pulse should be carefully chosen to excite only one dominant mode of Lamb wave. With findings from this paper, damage detection algorithms can be developed using the linear relationships between the damage size and the ultrasound energy diffusion coefficient or ultrasonic energy dissipation coefficient when a single dominant mode is excited.

## Figures and Tables

**Table 1:** Locations of the damages.

Damage Category Number	Location of Damage Center (mm, mm)
D1	(0, 0)
D2	(0, −60)

**Table 2:** Normalized ultrasound energy diffusion coefficient for damage category D1 (unit: %).

Damage Size (mm)	Sample Number			Average
	1	2	3	
0	100	100	100	100
Φ0.6	98.46	94.71	97.64	96.94
Φ0.8	98.15	92.76	96.73	95.88
Φ1.0	96.69	92.31	96.20	95.06
Φ1.2	94.22	91.46	95.29	93.67

**Table 3:** Normalized ultrasonic energy dissipation coefficient of damage category D1 (unit: %).

Damage Size (mm)	Sample Number			Average
	1	2	3	
0	100	100	100	100
Φ0.6	103.92	108.84	104.43	105.73
Φ0.8	104.74	111.72	106.36	107.61
Φ1.0	105.48	113.46	106.37	108.44
Φ1.2	106.22	114.79	107.32	109.44

**Table 4:** Normalized ultrasound energy diffusion coefficient of damage category D2 (unit: %).

Damage Size (mm)	Sample Number			Average
	1	2	3	
0	100	100	100	100
Φ0.6	98.97	96.46	97.46	97.63
Φ0.8	98.24	97.16	96.86	97.42
Φ1.0	98.48	95.73	96.03	96.74
Φ1.2	94.43	94.24	95.34	94.67

**Table 5:** Normalized ultrasonic energy dissipation coefficient of damage category D2 (unit: %).

Damage Size (mm)	Sample Number			Average
	1	2	3	
0	100	100	100	100
Φ0.6	104.75	105.44	103.84	103.67
Φ0.8	104.07	103.95	104.32	104.11
Φ1.0	103.26	105.51	105.23	104.66
Φ1.2	106.48	106.55	107.02	106.68

**Table 6:** Normalized displacements of different modes of Lamb waves.

Frequency/MHz	Mode of Lamb Waves			
	S_0_	S_1_	A_0_	A_1_
1.8	0.00087	-	0.0089	0.026
2.1	0.0023		0.0030	0.016
2.4	0.0026	0.016	0.0091	0.0028

**Table 7:** Group velocities of different modes of Lamb waves.

Frequency/MHz	Mode of Lamb Waves/ms^−1^			
	S_0_	S_1_	A_0_	A_1_
1.8	1898	-	3101	3726
2.1	2267	3559	3075	3243
2.4	3491	4391	3052	3108
